# Prognostic implications of statin intolerance in stable coronary artery disease patients with different levels of high-sensitive troponin

**DOI:** 10.1186/s12872-019-1152-x

**Published:** 2019-07-15

**Authors:** Jo-Jo Hai, Yuen-Kwun Wong, Chun-Ka Wong, Ka-Chun Un, Pak-Hei Chan, Chung-Wah Siu, Kai-Hang Yiu, Chu-Pak Lau, Hung-Fat Tse

**Affiliations:** 1 Cardiology Division, Department of Medicine, Queen Mary Hospital, The University of Hong Kong, Hong Kong, China; 2 Division of Cardiology, Department of Medicine, The University of Hong Kong-Shenzhen Hospital, Shenzhen, China; 3 The University of Hong Kong-Shenzhen Institute of Research and Innovation, Shenzhen, China; 4 Hong Kong-Guangdong Joint Laboratory on Stem Cell and Regenerative Medicine, the University of Hong Kong, Hong Kong, China

**Keywords:** Coronary artery disease, Statin intolerance, High sensitive troponin level

## Abstract

**Background:**

The prognostic implication of statin in tolerance (SI) in those with stable CAD remains unclear. We hypothesized that SI is of higher prognostic significance in stable CAD patients with elevated high-sensitive cardiac troponin I (hs-cTnI).

**Methods:**

A total of 952 stable CAD patients from the prospective Hong Kong CAD study who had complete clinical data, biomarker measurements and who were prescribed statin therapy were studied.

**Results:**

We identified 13 (1.4%) and 125 (13.1%) patients with complete and partial SI, respectively. At baseline, patients with SI were more likely to have diabetes mellitus and a higher hs-cTnI level, but no difference in LDL-C level compared with those without SI. After 51 months of follow-up, patients with SI had a higher mean LDL-C level than those without SI. A total of 148 (15.5%) patients developed major adverse cardiovascular events (MACEs). Both SI (HR 1.52, 95% CI 1.06–2.19, *P* = 0.02) and elevated hs-cTnI (HR 3.18, 95% CI 2.07–4.89, *P* < 0.01) were independent predictors of a MACE in patients with stable CAD. When stratified by hs-cTnI level, SI independently predicted MACE-free survival only in those with elevated hs-cTnI (HR 1.51, 95% CI 1.01–2.24, *P* = 0.04).

**Conclusions:**

SI independently predicted MACE in patients with stable CAD and high hs-cTnI, but not in those with low hs-cTnI. Hs-cTnI may be used to stratify stable CAD patients who have SI for intensive lipid-lowering therapy using non-statin agents.

**Electronic supplementary material:**

The online version of this article (10.1186/s12872-019-1152-x) contains supplementary material, which is available to authorized users.

## Background

Lowering of low-density lipoprotein cholesterol (LDL-C) is the cornerstone to the prevention and treatment of atherosclerotic cardiovascular diseases. International guidelines recommend the use of statin therapy for aggressive LDL-C reduction in patients with established coronary artery disease (CAD) for secondary prevention of cardiovascular events [1–5]. Although statins are widely available at low cost and generally well-tolerated, a substantial proportion of patients with established CAD receive suboptimal statin therapy [6–9]. Recent studies in an Asian population have shown than up to one-third to half of the patients with atherosclerotic cardiovascular disease receive suboptimal statin therapy [9, 10], with a consequent significantly lower achievement rate of target LDL-C level [11]. One of the major reasons for this is statin intolerance (SI) [6–9]. Currently, there is no widely accepted definition of SI, but it has been attributed to the adverse effects of statins that lead to treatment discontinuation, down-titration of dosage, repeated withholding of treatment or switching to an alternative lipid-lowering drug [6–9]. In a large cohort of Medicare beneficiaries in the United States that comprised patients with prior myocardial infarction, SI was associated with increased risk of cardiovascular events compared with those who adhered to the treatment [12]. Nonetheless there are limited data on the long-term prognostic implication of SI in patients with stable CAD. As a result, the appropriate management of patients with stable CAD and SI remains controversial [3–5].

In contrast to those with prior myocardial infarction, patients with stable CAD are more heterogeneous for their future risk of cardiovascular events. Although it is difficult to stratify this group of patients using clinical parameters, studies have shown that an elevated high-sensitive cardiac troponin I (hs-cTnI) level is associated with the severity and long-term outcomes of stable CAD [13–15]. Indeed, recent follow-up data from the West of Scotland Coronary Prevention Study (WOSCOPS) have shown that statin therapy provides more benefit to individuals with a high level of hs-cTnI than to those with a low level [16]. We hypothesized that SI is of higher prognostic significance in stable CAD patients with elevated hs-cTnI. In this study, we sought to investigate the cardiovascular outcomes of patients with stable CAD, with and without SI stratified by hs-cTnI level.

## Methods

### Study design

The Hong Kong CAD study is a single-center prospective observational study of patients with stable CAD and was approved by the local Institutional Review Board [6–8]. Patients who were > 18 years old and had stable coronary artery disease newly diagnosed by conventional angiography at Queen Mary Hospital between 1st December 2003 and 30th November 2014 were recruited. Those with impaired left ventricular ejection fraction < 50%, moderate or severe valvular heart disease, acute coronary syndrome or who were unable to or refused to provide informed consent were excluded. Clinical information on admission including age, gender, past medical conditions, family history, cardiac biomarker levels, electrocardiographic findings, echocardiographic data, medications prescribed, coronary angiography reports and revascularization results were recorded at baseline. All patients were prescribed statin prior to admission for coronary angiography and continued indefinitely after the diagnosis of CAD was confirmed. Data on their subsequent clinical course including medication usage, dosage and adverse effects were retrieved from the comprehensive electronic medical system of the Hong Kong Hospital Authority as previously described [17–19]. Patients with incomplete clinical records, insufficient plasma sample for analysis, or those who refused or were not prescribed a statin on discharge were excluded.

### Definitions of statin intolerance

In this study, SI was defined as an inability to continue use of a statin, either because of side effects, or because of elevated liver enzymes or creatine kinase that were sufficiently abnormal to cause concern [7]. Cases of SI were retrospectively ascertained using a standardized protocol (Additional file 1). In brief, patients were considered completely SI if they were unable to tolerate the initial dose of one statin and any dose of another statin [7]. Patients were considered partially SI if they were unable to tolerate at least one statin at one dose [7].

### Measurement of cardiac biomarker

All subjects had plasma stored at − 80 °C at baseline. Serum level of hs-cTnI was retrospectively determined for this study after completion of patient recruitment, using a Chemoluminescent Microparticule ImmunoAssay (Architect i1000SR Abbott®, Paris, France) according to the manufacturer’s protocol. The level of detection was 1.2 ng/L and the 99th percentile value of serum hs-cTnI level in male and female control subjects was 8.5 ng/L and 7.6 ng/L, respectively [17–19]. Based on a baseline sample in 20 patients, the intra-assay coefficient of variations was 2.5%.

### Clinical outcomes

Patients were monitored until the diagnosis of a cardiovascular endpoint, death or last clinical visit. The primary endpoint of this study was new-onset major adverse cardiovascular event (MACE). We adjudicated new-onset MACE based on the *International Classification of Diseases, Ninth Revision (ICD-9):* acute myocardial infarction *(ICD-9 410)*, acute coronary syndrome *(ICD-9 411.1)*, stroke *(ICD-9 430, 431, 433, 434, 436)*, peripheral vascular disease *(ICD-9 443.9)* and cardiovascular death *(death certificate ICD-9 410–447)*. Information on discharge diagnosis and date of events were confirmed by reviewing electronic records of the Hong Kong Hospital Authority by two independent cardiologists [17–19]. For patients who died during follow-up, the main cause and date of death were obtained from the Hong Kong Death Registry.

### Statistical analysis

Results are expressed as mean ± standard deviation or number and percentage as appropriate. Normality assumption was evaluated using the Kolmogorov-Smirnov test. The biomarker hs-cTnI was logarithmically transformed before analysis. Patient age was categorized as < 65 or ≥ 65 years. The optimal cut-off value for hs-cTnI was determined using the Youden J index. Cox proportional hazards regression was performed to examine the association of age, clinical risk factors, statin intolerance and hs-cTnI with MACE. Hazard ratios (HRs) and 95% confidence intervals (CIs) were estimated for MACE. Additional variables including advanced age, male sex, active smoking, hypertension and diabetes were selected using minimized Akaike information criterion to build the multivariate model [20]. Kaplan-Meier survival curve and log-rank test were used to assess the relationship between SI, hs-cTnI and MACE. A two-sided *P*-value < 0.05 was considered statistically significant. All analyses were performed using statistical software packages SPSS (version 19; SPSS, Chicago, IL), STATA (version 14.0) and R-programming language (version 3.5.2).

## Results

### Baseline characteristics

Our cohort consisted of 1202 patients who had stable CAD diagnosed by conventional coronary angiography. Patients were excluded if they had missing clinical data (*n* = 33) or biomarker measurement (*n* = 214), or refused or were not prescribed a statin on discharge (*n* = 3). A total of 952 patients were retained in the analysis and their clinical demographic features are summarized in Table 1. We identified 138 (14.5%) patients who suffered either complete SI [13 (1.4%)] or partial SI [125 (13.1%)]. Two patients concomitantly received ezetimibe at baseline. Among those with SI, only two (1.5%) were due to significant elevation in creatine kinase, one (0.7%) was due to significant elevation in liver enzymes, and the remainder were due to symptoms attributed to statin use. Patients with complete SI were treated with ezetimibe (*n* = 1), fibrate (*n* = 3), or no lipid lowering therapy (*n* = 9). Patients with partial SI were treated with a reduced dose of statin (*n* = 7) or an alternative statin (*n* = 100), and add-on ezetrimibe (*n* = 7) or fibrate (*n* = 11).

**Table 1:** Clinical characteristics of patients with and without statin intolerance

	All *N* = 952	Statin Intolerance *N* = 138	No Statin Intolerance *N* = 814	*P*-value
Age, years	66 ± 11	67 ± 10	66 ± 11	0.79
Age ≥ 65 years, n (%)	558 (58.6)	81 (58.7)	477 (58.6)	0.98
Male, n (%)	696 (73.1)	99 (71.7)	597 (73.3)	0.70
Current smoker, n (%)	160 (16.8)	25 (18.1)	135 (16.6)	0.66
Hypertension, n (%)	716 (75.2)	107 (77.5)	609 (74.8)	0.49
Diabetes mellitus, n (%)	336 (35.3)	61 (44.2)	275 (33.8)	0.02
Type of statin at baseline				
*Simvastatin, n (%)*	537 (56.4)	82 (59.4)	455 (55.9)	0.50
*Atorvastatin, n (%)*	179 (18.8)	21 (15.2)	158 (19.4)	
*Rosuvastatin, n (%)*	236 (24.8)	35 (25.4)	201 (24.7)	
Statin dose at baseline (simvastatin dose equivalent)				
*10 mg, n (%)*	227 (23.8)	30 (21.7)	197 (24.2)	0.91
*20 mg, n (%)*	365 (38.3)	56 (40.6)	309 (38.0)	
*40 mg, n (%)*	254 (26.7)	36 (26.1)	218 (26.8)	
*80 mg, n (%)*	106 (11.1)	16 (11.6)	90 (11.1)	
Baseline lipid profile*				
*Total cholesterol, mmol/L*	4.1 ± 1.0	4.2 ± 1.1	4.0 ± 1.0	0.08
*Triglyceride, mmol/L*	1.5 ± 1.0	1.7 ± 1.2	1.5 ± 1.0	< 0.01
*HDL-C, mmol/L*	1.2 ± 0.4	1.2 ± 0.5	1.2 ± 0.3	0.78
*LDL-C, mmol/L*	2.1 ± 0.8	2.2 ± 0.9	2.1 ± 0.8	0.71
*LDL-C < 1.8 mmol/L, n (%)*	359 (37.7)	49 (35.5)	310 (38.1)	0.56
*Non-HDL-C, mmol/L*	2.9 ± 1.0	3.0 ± 1.0	2.8 ± 1.0	0.09
Ln hs-cTnI, pg/ml	2.2 ± 1.4	2.6 ± 1.6	2.1 ± 1.3	< 0.01
Type of statin at follow-up				
*Simvastatin, n (%)*	626 (65.8)	93 (67.4)	533 (65.5)	0.41
*Atorvastatin, n (%)*	145 (15.2)	16 (11.6)	129 (15.9)	
*Rosuvastatin, n (%)*	181 (19.0)	29 (21.0)	152 (18.7)	
Statin dose at follow-up (simvastatin dose equivalent)				
*10 mg, n (%)*	280 (29.4)	39 (28.3)	241 (29.6)	0.84
*20 mg, n (%)*	378 (39.7)	55 (39.9)	323 (39.7)	
*40 mg, n (%)*	203 (21.3)	28 (20.3)	175 (21.5)	
*80 mg, n (%)*	91 (9.6)	16 (11.6)	75 (9.2)	
Other lipid lowering therapy at follow-up				
*Ezetimible, n (%)*	32 (3.4)	8 (5.8)	24 (3.0)	0.09
*Fibrate, n (%)*	82 (8.6)	14 (10.1)	68 (8.4)	0.49
Lipid profile at follow-up				
*Total cholesterol, mmol/L*	3.7 ± 0.8	3.9 ± 1.0	3.7 ± 0.7	< 0.01
*Triglyceride, mmol/L*	1.4 ± 0.9	1.6 ± 1.1	1.3 ± 0.8	< 0.01
*HDL-C, mmol/L*	1.2 ± 0.4	1.2 ± 0.4	1.2 ± 0.4	0.09
*LDL-C, mmol/L*	1.9 ± 0.6	2.0 ± 0.8	1.9 ± 0.6	0.01
*LDL-C < 1.8 mmol/L, n (%)*	412 (43.3)	51 (37.0)	361 (44.4)	0.11
*Non-HDL-C, mmol/L*	2.5 ± 0.7	2.7 ± 0.9	2.5 ± 0.7	< 0.01

*Baseline lipid profile was taken after the diagnosis of CAD was confirmed by coronary angiography

Abbreviations: *HDL-C* high density lipoprotein cholesterol, *hs-cTnl* high-sensitive cardiac troponin I, *Ln* natural logarithm, *LDL-C* low density lipoprotein cholesterol

Of note, patients with SI were more likely to have diabetes mellitus [61 (44.2%) vs. 275 (33.8%), *P* = 0.02] and have a higher hs-cTnI (2.6 ± 1.6 vs. 2.1 ± 1.3 ng/L, *P* < 0.01) at baseline than those without SI. Nevertheless there were no significant differences in LDL-C level, proportion of patients with LDL-C < 1.8 mmol/L, type or dose of statin between patients with or without SI (all *P > 0.05*. Table 1). At follow-up, although the total cholesterol (3.9 ± 1.0 vs. 3.7 ± 0.7 mmol/L, *P* < 0.01) and mean LDL-C level (2.0 ± 0.8 vs.1.9 ± 0.6 mmol/L, *P* = 0.01. Table 1) was significantly higher in patients with than those without SI, there were no significant differences in the proportion of patients with LDL-C < 1.8 mmol/L, the type of dose of statin between patients with and without SI (all *P* > 0.05, Table 1).

### Clinical outcomes

**Fig. 1:**
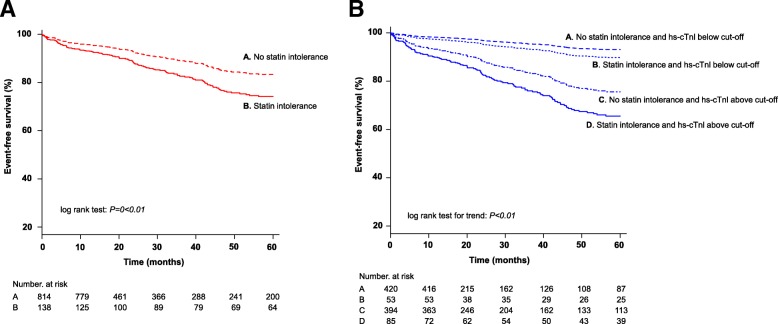
**a** Kaplan-Meier curves comparing event-free survival between patients with and without statin intolerance. **b** Kaplan-Meier curves comparing event-free survival between patients with and without statin intolerance stratified by hs-cTnI

After a mean follow-up of 51 ± 42 months, 148 (15.5%) patients developed a MACE, including 54 (5.7%) acute coronary syndrome; 19 (2.0%) stroke; 9 (0.9%) peripheral vascular events and 66 (6.9%) cardiovascular deaths. Clinical characteristics of CAD patients with and without MACE are summarized in Table 2. Stable CAD patients with MACE on follow-up were older, and a higher proportion were male or had hypertension or diabetes compared with those without MACE (Table 3, all *P < 0.05*). Moreover, patients who developed a MACE were more likely to have SI [41 (27.7%) vs. 97 (12.1%), *P* < 0.01] than those who did not (Table 2). Kaplan-Meier MACE-free survival showed a significant difference between stable CAD patients with and without SI (log-rank *P* < 0.01. Figure 1a).

**Table 2:** Clinical characteristics of patients with and without MACE

	With MACE *N* = 148	Without MACE *N* = 804	*P*-value
Age, years	72 ± 9	66 ± 11	< 0.01
Age ≥ 65 years, n (%)	120 (81)	438 (54)	< 0.01
Male, n (%)	98 (66)	598 (74)	0.040
Current smoker, n (%)	29 (20)	131 (16)	0.32
Hypertension, n(%)	129 (87)	587 (73)	< 0.01
Diabetes mellitus, n (%)	86 (51)	260 (32)	< 0.01
Baseline lipid profile*			
*Total cholesterol, mmol/L*	4.2 ± 1.1	4.0 ± 1.0	0.17
*Triglyceride, mmol/L*	1.5 ± 1.0	1.5 ± 1.0	0.71
*HDL-C, mmol/L*	1.2 ± 0.5	1.2 ± 0.3	0.51
*LDL-C, mmol/L*	2.2 ± 1.0	2.1 ± 0.8	0.13
*LDL-C < 1.8 mmol/L, n (%)*	56 (37.8)	303 (37.7)	0.97
*Non-HDL-C, mmol/L*	3.0 ± 1.1	2.8 ± 1.0	0.10
Follow-up lipid profile			
*Total cholesterol, mmol/L*	3.7 ± 0.8	3.8 ± 0.8	0.17
*Triglyceride, mmol/L*	1.4 ± 0.9	1.4 ± 0.9	0.86
*HDL-C, mmol/L*	1.2 ± 0.4	1.2 ± 0.4	0.16
*LDL-C, mmol/L*	1.9 ± 0.7	1.9 ± 0.6	0.65
*LDL-C < 1.8 mmol/L, n (%)*	69 (46.6)	343 (42.7)	0.37
*Non-HDL-C, mmol/L*	2.5 ± 0.7	2.5 ± 0.7	0.45
Statin intolerance, n (%)	41 (27.7)	97 (12.1)	< 0.01
Ln hs-cTnI, pg/ml	3.1 ± 1.6	2.0 ± 1.2	< 0.01

*Baseline lipid profile was taken after the diagnosis of CAD was confirmed by coronary angiography

Abbreviations: *HDL-C* high density lipoprotein cholesterol, *hs-cTnl* high-sensitive cardiac troponin I, *Ln* natural logarithm, *LDL-C* low density lipoprotein cholesterol, *MACE* major adverse cardiovascular events

**Table 3:** Multivariable Cox regression analysis predicting MACE in patients with stable CAD

	HR (95% CI)	*P*-value
Age ≥ 65 years	2.72 (1.79–4.15)	< 0.01
Hypertension	1.37 (0.83–2.27)	0.22
Diabetes mellitus	1.26 (0.90–1.77)	0.18
hs-cTnI	3.18 (2.07–4.89)	< 0.01
Statin intolerance	1.52 (1.06–2.19)	0.024

Abbreviations: *CI* confidence interval, *HR* hazard ratio, *hs-cTnl* high-sensitive cardiac troponin I. hs-cTnl are at level above 5.2 pg/ml

### Effect of hs-cTnI level on MACE

As shown Table 1, hs-cTnI level was significantly higher in CAD patients with SI than those without (2.6 ± 1.6 vs. 2.1 ± 1.3 ng/l, *P* < 0.01*).* Moreover, hs-cTnI level was significantly higher in CAD patients with MACE than those without (3.1 ± 1.6 vs. 2.1 ± 1.2 ng/l, *P* < 0.01*).* The cut-off value of hs-cTnI for MACE derived from the Younden J index was 5.2 ng/l. Multivariable Cox regression analysis showed that both SI (HR 1.52, 95% CI 1.06–2.19, *P* = 0.02) and elevated hs-cTnI level (HR 3.18, 95% CI 2.07–4.89, *P* < 0.01) were independent predictors of MACE in patients with stable CAD (Table 3). Nevertheless, when stratified by hs-cTnI level, SI independently predicted worse MACE-free survival in patients with elevated hs-cTnI (HR 1.51, 95% CI 1.01–2.24, *P* = 0.04), but not in patients without (HR 1.40, 95% CI 0.55–3.52, *P* = 0.48. Table 4). The MACE-free survival was significantly worse in stable CAD patients with both SI and elevated baseline hs-cTnI than those without SI or elevated hs-cTnI (log-rank *P* < 0.01. Figure 1b).

**Table 4:** Multivariable Cox regression analysis predicting MACE in patients with stable CAD stratified by hs-cTnI

	hs-cTnl below cut-off		hs-cTnl above cut-off	
	HR (95% CI)	*P*-value	HR (95% CI)	*P*-value
Age ≥ 65	2.70 (1.15–6.34)	0.022	2.75 (1.69–4.47)	< 0.01
Hypertension	1.26 (0.49–3.24)	0.63	1.44 (0.79–2.63)	0.24
Diabetes	1.05 (0.47–2.36)	0.90	1.29 (0.89–1.87)	0.19
Statin intolerance	1.40 (0.55–3.52)	0.48	1.51 (1.01–2.24)	0.044

Abbreviations: *CI* confidence interval, *HR* hazard ratio, *hs-cTnl* high-sensitive cardiac troponin I

## Discussion

The prevalence of SI depends strongly on the definition employed, setting of treatment and method of case ascertainment. Although SI has been diagnosed in only 3 to 5% of subjects in randomized controlled trials, its reported prevalence has been as high as 10–30% in clinical registries [6–9]. Interestingly, the incidence of statin-related adverse effects also varies across different populations, ranging from 2% in Italy and Spain to over 10% in Japan, Canada, United Kingdom and United States [9, 21]. It is believed that both genetic and cultural factors play a role in the observed differences in SI in different populations.

Pharmacokinetic studies have shown that Asians achieve a higher plasma level of statin compared with Caucasians [22–24]. Based on these findings, the United States Food and Drug Administration has listed Asian ethnicity as a risk factor for statin-related adverse effects, and recommend a lower dose of statin for this population [22, 23]. In this study, we observed that ~ 15% of Chinese patients with stable CAD suffered from SI, similar to that reported in Western registries. However, only 1.4% of our patients were regarded as having complete SI and in whom statin therapy was terminated over a mean of 51 months of follow-up. These findings imply that complete SI is rare, and that statin is generally well-tolerated in our Chinese patients with stable CAD.

This is the first study to investigate the impact of SI on long-term clinical outcomes in a cohort of patients with stable CAD. Although we have shown that SI is an independent predictor of MACE in patients with stable CAD and high hs-cTnI, it did not predict clinical outcomes in those with low hs-cTnI. This finding demonstrates that the risk of future cardiovascular events is not uniform among CAD patients with SI. In this study, we observed only a small, albeit statistically significant, difference in LDL-C level during follow-up in patients with SI compared with those without. It is likely because complete SI was rare in our cohort and those patients with partial SI continued statin therapy. Despite this small difference in LDL-C, there was a remarkable 50% increase in cardiovascular events among our CAD patients with SI. As pointed out by other researchers, SI does not necessarily lead to treatment discontinuation, but its effect of repeated withholding and re-initiation, as well as up- and down-titration of statin therapy may impose an adverse effect on the clinical outcomes of the patients [6–9]. Importantly, our results confirmed the previous findings [13–15] of the predictive role of hs-cTnI for MACE in stable CAD patients and extended it prognostic role in those CAD patients with SI. Indeed, stable CAD patients who had SI and elevated hs-cTnI had significantly worse MACE-free survival than other patients with stable CAD. Therefore, SI appeared to have stronger prognostic implication for those with high hs-cTnI than those with low hs-cTnI.

The findings of this study have potentially important clinical implications for the management of stable CAD patients with SI. In the latest published guideline by the American Heart Association, alternative non-statin therapies, such as ezetimibe and proprotein convertase subtilisin/kexin type 9 (PCSK9) inhibitors, should be considered in a ‘very high-risk’ subgroup of CAD patients, defined by the presence of multiple prior cardiovascular events, who have suboptimal LDL-C lowering despite maximally tolerated statin therapy [5]. Nevertheless there is no clear guidance on the management of stable CAD patients with SI. Our results suggest that more aggressive non-statin lipid lowering treatment should be considered in CAD patients with elevated hs-cTnI and SI, even in the absence of prior myocardial infarction. Recently, PCSK9 inhibitors have been shown to be effective in lowering LDL-C in patients with SI and to robustly reduce cardiovascular events in a broad spectrum of CAD patients with and without prior myocardial infarction [25, 26]. Accordingly, stable CAD patients with elevated hs-cTnI and suboptimal statin therapy due to SI should be potential candidates for PCSK9 inhibitors due to their higher risk of future cardiovascular events.

### Limitations

First, this was a single-center cohort study that included only CAD patients treated with statin, which inadvertently resulted in selection bias over patients who could tolerate the initiation of statin therapy. As a result, we might have underestimated the prevalence of SI in our population. Second, some patients in this study were recruited over 15 years ago, when the benefit of high-dose statin and low LDL-C in stable CAD was less well-established. Since stable patients are often continued with the same statin dose after initial prescription [9, 10], a significant proportion of patients in this study remained on low dose statin. In fact, only 43% of patients without SI in this study achieved a target LDL-C level of < 1.8 mmol/L on follow-up, which was similar to those observed in other Asian countries including Taiwan and Japan [9, 10]. As the latest guidelines recommended titration of both statin and non-statin therapies to achieve a 30–49% reduction in LDL-C [5], it remains unclear whether the adoption of this approach will alter the results of this study. Third, retrospective assessment of hs-cTnI in stored serum samples using immunoassays may result in random measurement errors. It is because the long-term stability of cTnI in frozen sample has remained debatable [27–30], together with the presence of heterophile or anti-TnI antibodies, may lead to erroneous hs-cTnI values [31]. Nevertheless, in a recent study that assessed cTnI levels using the Architect STAT hs-cTnI assay (Abbott Laboratories, IL, USA) in serum samples that were stored for 0.8 to 82.0 months, the mean cTnI value in the quartile of samples with the longest storage time did not differ significantly from those with the shortest storage time, suggesting that the magnitude of cTnI degradation over a long period of storage time may be small compared with the actual cTnI value [32]. Moreover, how the presence or absence of antibodies interfere with the prognostic significance of hs-cTnI values remain unknown [31]. Despite these limitations, hs-cTnI has been shown to predict clinical outcomes in a variety of cardiovascular conditions [13–15], which is consistent with the findings of this study. Before the development of a simple and low-cost test that is negatively influenced by those antibodies, our findings is still of significant value as hs-cTnI measured by immunoassays is widely available in the clinical setting.

## Conclusion

The results of this study showed that SI independently predicted MACE in patients with stable CAD and hs-cTnI > 5.2 ng/L, but not in those with low hs-cTnI. Our findings suggested that a cut-off value of hs-cTnI > 5.2 ng/L is useful to stratify patients with stable CAD to more aggressive non-statin lipid lowering treatment.

## Additional file

Additional file 1: Protocol for identification of statin intolerance. (DOCX 15 kb)

## Data Availability

The datasets used and / or analyzed during the current study are available from the corresponding author on reasonable request.

## Abbreviations

CAD: Coronary artery disease
CI: Confidence interval
HR: Hazard ratio
Hs-cTnI: High-sensitive cardiac troponin I
LDL-C: Low-density lipoprotein cholesterol
MACE: Major adverse cardiovascular event
SI: Statin intolerance
WOSCOP: West of Scotland coronary prevention study
