# Supplementary material for: Prognostic implications of statin intolerance in stable coronary artery disease patients with different levels of high-sensitive troponin
Source: BMC Cardiovasc Disord. 2019 Jul 15;19:168. doi: 10.1186/s12872-019-1152-x (PMC6633694; doi:10.1186/s12872-019-1152-x)
Supplement: Supplementary file 1 — Protocol for identification of statin intolerance. (DOCX 15 kb) [file 12872_2019_1152_MOESM1_ESM.docx]

**Protocol for identification of statin intolerance**

Patients who were indicative of intolerant to statin will be identified for the assessment of primary outcome:

a. For Former Statin Users

Patient records would be checked for any documented adverse event(s) following the initiation or up-titration of statin.

Patients are considered to be complete statin-intolerant if they are intolerant to the starting dose of one statin AND any dose of another statin AND NOT attributable to establish predispositions e.g. Untreated hypothyroidism etc.

Patients are considered to be partial statin-intolerant if they are Intolerant to some statins at some doses.

b. For Current Statin Users:

I. After initiation or up-titration of statin, those who have abnormal test findings of either i) creatine Kinase (CK) >10 x upper limit of normal (ULN) or ii) liver enzymes elevation of > 3 x ULN, that improved or resolved with 1) dose reduction; 2) changing to alternate-day statin; or 3) switching to another statin, they will be regarded as partial statin intolerant;

II. Those who have i) abnormal test findings of CK >3 and <10 x ULN AND ii) prescription changes of either 1) dose reduction; 2) changing to alternate-day statin; or 3) switching to another statin, they will be defined as partial statin intolerant;

III. Those who only have prescription changes of 1) dose reduction; or 2) changing to alternate-day statin; or 3) switching to another statin, their medical records would be checked for any documented adverse event(s) following the initiation or up-titration of statin. If the changes are caused by the experience of adverse events, they will be defined as partial statin intolerant.

IV. Those who ultimately received less than an equivalent dose of statin to simvastatin 40mg daily, their patient record would be checked for any documented adverse event(s) following the initiation or up-titration of statin. If any adverse event(s) had been documented, the patient would be defined as partial statin-intolerant. If there was no documented adverse event(s), they would be contacted via telephone to understand if there was any adverse event(s) that precluded them from taking high-dose statin.

V. Those whose LDL-C level <1.8 mmol/L, their patient record would be checked for any documented adverse event(s) following the initiation or up-titration of statin. If any adverse event(s) had been documented, the patient would be defined as partial statin-intolerant. If there was no documented adverse event(s), they would be contacted via telephone to understand their adherence of statin. Those who are #non-adherent would be further verified if there was any adverse event(s) that precluded them from adhering to statin.

#Non-adherence was defined as taking <20% of their prescribed statin dose. It would be estimated according to the number of missing dose in the past one month and intentional reduction of statin dosage by the patient.
